# Magnetic Properties of Magnetic Nanoparticles for Efficient Hyperthermia

**DOI:** 10.3390/nano5010063

**Published:** 2015-01-09

**Authors:** Ihab M. Obaidat, Bashar Issa, Yousef Haik

**Affiliations:** 1 Department of Physics, United Arab Emirates University, Al-Ain 15551, United Arab Emirates; E-Mail: b.issa@uaeu.ac.ae; 2 Department of Mechanical Engineering, United Arab Emirates University, Al-Ain 15555, United Arab Emirates; E-Mail: yhaik@uaeu.ac.ae; 3 Center for Research Excellence in Nanobiosciences, University of North Carolina at Greensboro, Greensboro, NC 27412, USA

**Keywords:** magnetic nanoparticles (MNPs), hyperthermia, power dissipation, curie temperature, anisotropy

## Abstract

Localized magnetic hyperthermia using magnetic nanoparticles (MNPs) under the application of small magnetic fields is a promising tool for treating small or deep-seated tumors. For this method to be applicable, the amount of MNPs used should be minimized. Hence, it is essential to enhance the power dissipation or heating efficiency of MNPs. Several factors influence the heating efficiency of MNPs, such as the amplitude and frequency of the applied magnetic field and the structural and magnetic properties of MNPs. We discuss some of the physics principles for effective heating of MNPs focusing on the role of surface anisotropy, interface exchange anisotropy and dipolar interactions. Basic magnetic properties of MNPs such as their superparamagnetic behavior, are briefly reviewed. The influence of temperature on anisotropy and magnetization of MNPs is discussed. Recent development in self-regulated hyperthermia is briefly discussed. Some physical and practical limitations of using MNPs in magnetic hyperthermia are also briefly discussed.

## 1. Introduction

### 1.1. Localized Magnetic Hyperthermia

Magnetic hyperthermia is the field of treating cancer by supplying heat to tumor cells using magnetic nanoparticles (MNPs) and an alternating magnetic field. This method could be promising to treat small or deep-seated tumors. Magnetic hyperthermia using MNPs is a multidiscipline research field which requires the involvement of physics, chemistry, material science and medical science. This technique, which started in 1957 [1], where maghemite nanoparticles (γ-Fe_2_O_3_) were used, is based on the observation that tumor cells can be destroyed by heating the cells for a duration of time to temperature between 43 and 46 °C while healthy cells are less affected [2,3]. The heating process is enabled by the application of an alternating magnetic field of suitable amplitude and frequency. One of the major issues that is being investigated in magnetic hyperthermia is the reduction of the amount of MNPs that can be used in living organs [2,3]. In order to achieve this goal, the power dissipation or heating efficiency of MNPs should be enhanced. Several factors influence the heating efficiency, such as the amplitude and frequency of the external alternating magnetic field, magnetic anisotropy, magnetization, particle-particle interactions, as well as the size and size distribution of the MNPs. There are several excellent reviews that discuss magnetic hyperthermia using MNPs [4,5,6].

There are also several excellent reviews on the physics of heating efficiency using magnetic nanoparticles in magnetic hyperthermia [7,8,9]. In this short review we focus on the physical and magnetic properties of MNPs that are related to heating efficiency in magnetic hyperthermia. We only discuss selective recent reports that display interesting results that could influence magnetic properties for magnetic hyperthermia.

### 1.2. Main Properties of MNPs

Other than their intense applications in data storage devices [10,11], MNPs have several other technological applications in biomedicine [12,13] such as magnetic resonance imaging, drug delivery and magnetic hyperthermia.

Magnetic properties of nanoparticles (NPs) are dominated by two main features [14]; finite-size effects (single-domain, multi-domain structures and quantum confinement) and surface effects, which results from the symmetry breaking of the crystal structure at the surface of the particle, oxidation, dangling bonds, surface stain, *etc*. Surface effects become significant as the particle size decreases because the ratio of the number of surface atoms to the core atoms increases. It is well established that several magnetic properties such as magnetic anisotropy, magnetic moment per atom, Curie temperature, and the coercivity field of NPs can be different than those of a bulk material [14,15]. In most medical applications, the preferred size of the nanoparticles is typically around 10–50 nm. In this range of sizes, usually a nanoparticle becomes a single magnetic domain (for minimization of its magnetic energy) and shows superparamagnetic behavior when the temperature is above a certain temperature called the blocking temperature. In the superparamagnetic state, a nanoparticle possesses a large magnetic moment and behaves like a giant paramagnetic atom with a fast response to applied magnetic fields with negligible remanence and coercivity. For hyperthermia applications, MNPs must have high saturation magnetization, *M*_s_ values. High *M*_s_ values will result in large thermal energy dissipation in the tumor cells. On the other hand, large *M*_s_ values give more control on the movement of the MNPs in the blood using external magnetic field. However, it is important to understand that in order to apply MNPs in hyperthermia, the NPs must satisfy two main conditions: they should have large heating power, and they should have good stability. For good stability, MNPs are preferred to be superparamagnetic. In the absence of an applied magnetic field, the superparamagnetic NPs lose their magnetism at temperatures above the blocking temperature. This enables the particles to avoid aggregation and maintain their colloidal stability. On the other hand, dipolar interactions between MNPs become very small as the particles’ sizes become very small. This is the case because the dipole-dipole interaction energy scale as *r*^6^ (*r* is the inter-particle distance between particles). Reducing the dipolar interactions will minimize particle aggregation in the existence of applied magnetic field. However, with regard to the other condition, superparamagnetic NPs might not be the best choice. In [16], it was reported that heating power was maximized in large ferromagnetic NPs with low anisotropy. In addition, as reported in [17], the optimum size for the maximum power loss varies with the amplitude of the applied magnetic field. Hence, the choice between superparamagnetic and ferromagnetic NPs for hyperthermia is not a simple task where several experimental conditions should be considered.

There are five main factors that determine the magnetic properties of nanoparticles. These are: (a) the geometrical properties of the nanoparticles; (b) magnetic interactions that occur inside the nanoparticle (intra-particle interactions); (c) particle-particle magnetic interactions (inter-particle interactions); (d) magnetic interactions that occur between the nanoparticles and the matrix material; and (e) particle-applied magnetic field interactions. The geometrical properties of the nanoparticles include: (a) sizes of particles; (b) shapes of particles; (c) distributions of sizes; and (d) distributions of anisotropy axes. Interactions inside a magnetic nanoparticle (intra-particle interactions) include: (a) those inside the domain of a ferromagnetic particle, where the magnetic moments of atoms interact via the exchange interaction; (b) those inside the domain of a ferrimagnetic particle, where the magnetic moments of atoms interact via the super-exchange interaction; (c) interaction of moments inside a multi-domain particle with each other; and (d) interaction of moments at the surface of a nanoparticle with moments of the interior of the particle. Interactions between magnetic nanoparticles (inter-particle interactions) include: (a) dipolar (dipole-dipole) interactions between the net magnetic moments of the particles; (b) direct exchange interactions between moments at the surfaces of touching particles; and (c) super-exchange interactions between non-touching particles such as magnetic particles which are placed in an insulating matrix. The interactions between particles and the magnetic field include: (a) interaction between magnetic moments of the magnetic domains and the applied magnetic field; and (b) interactions between moments at the surface of a nanoparticle with the applied magnetic field. In any particular sample of nanoparticles, some or even all of these factors and interactions might exist simultaneously. It is not simple to separate the geometrical roles from the interaction roles. The most dominant interaction between particles is the dipolar interaction.

## 2. Physics of Heating of MNPs

### 2.1. Relaxation of Magnetic Moment

The spin-orbital interactions of the electrons in the NP produce magnetic anisotropy. For isolated systems, the magnetic anisotropy is responsible for keeping the spins in a particular direction. Because atomic orbitals mainly have non-spherical shapes, they prefer to align in a specific crystallographic direction which is called the easy direction. Because in materials with large magnetocrystalline anisotropy, the atomic spin and orbital angular moments are strongly coupled, magnetization prefers to align along the easy direction. Energy is needed in order to rotate the magnetization away from the easy direction. This energy is called the anisotropy energy. In the case with uniaxial anisotropy, the anisotropy energy per particle is given by [18]:

(1) $$E=K\;V\;sin^{2}\theta +higher\;order\;terms$$

where K is the anisotropy constant (it includes all sources of anisotropy), *V* is the volume of the particle, and θ is the angle between the particle magnetization and the easy magnetization axis of the particle. The higher order terms can be ignored since they are very small compared with the first term. This anisotropy energy with uniaxial anisotropy has one easy axis with two energy minima separated by the energy maximum, KV. As can be seen from Equation (1), the anisotropy energy directly depends on the particle size and on the anisotropy constant. For a fixed K, as *V* decreases, E decreases. At very small particle sizes, the particle will prefer to have only one magnetic domain and thus called single-domain NP. At this small size, the anisotropy energy might become smaller than the thermal energy,$\;E_{\mathrm{th}}=\;k_{B}T$ ($k_{B}$ is the Boltzmann constant). Once this happens, the particle magnetic moment starts to rotate freely in all directions leading to zero net magnetization in the absence of an external magnetic field. If the flipping of magnetic moment occurs while the particle orientation is fixed, then the relaxation time of the moment of a particle is called the Néel relaxation time, $\tau_{N}$ and is given by [8,9,19,20]:

(2) $$\tau_{N}=\frac{\tau_{o}}{2}\sqrt{\frac{\pi k_{B}T}{K_{\mathrm{eff}}V}}\exp\left(\frac{K_{\mathrm{eff}}V}{k_{B}T}\right)$$

where $K_{\mathrm{eff}}$ is the effective anisotropy and the factor τ0≈10−13−10−9 s [4,21].

When measuring the magnetization of a superparamagnetic NP, we define $\tau_{m}$ to be the measurement time. If $\tau_{m}\gg \tau_{N}$, the magnetization of the NP will flip several times during the measurement giving zero average magnetization. In this case, the NP is said to be in the superparamagnetic state. If $\tau_{m}\ll \tau_{N}$, the magnetization will not have enough time to flip during the measurement and will be blocked at the initial non-zero value at the beginning of the measurement. In this case, the NP is said to be in the blocked state. The transition between the superparamagnetic state and the blocked state occurs when $\tau_{m}=\tau_{N}$ [18]. If in an experiment, the measurement time is kept constant while the temperature was varied, the transition between superparamagnetic and blocked states is obtained as a function of temperature. The temperature at which this transition occurs is called the blocking temperature, *T*_b_. Thus, at *T*_b_ the measurement time will be equal to the Neel relaxation time, $\tau_{m}=\tau_{N}$. As mentioned above, the size of the particle is crucial in determining the blocking temperature. The blocking temperature also depends on other factors such as particle-particle interactions. Superparamagnetic behavior of non-interacting single-domain particles occurs at temperatures larger than the blocking temperature. It is important to note that mainly all superparamagnetic particles are single-domain particles, but not all single-domain particles are superparamagnetic. In the superparamagnetic state, magnetization disappears as long as no magnetic field is applied.

If the particle itself rotates while the flipping of the particle’s moment occurs, moment relaxation is called the Brownian relaxation mechanism, $\tau_{B}$ and is given by [8,9,22]:

(3) $$\tau_{B}=\frac{3\;V_{H}\;\eta }{k_{B\;}T}$$

where η is the viscosity of the liquid containing the particles and $V_{H}$ is the hydrodynamic volume of the particle. Because of particle coating, absorbed surfactants or interaction with the fluid, $V_{H}$ is larger than the original volume of the particle, V. The effective magnetic relaxation time, $\tau_{\mathrm{eff}}\;$ is then given by:

(4) $$\frac{1}{\tau_{\mathrm{eff}}}=\frac{1}{\tau_{N}}+\;\frac{1}{\tau_{B}}$$

It can be seen that it is the shorter relaxation time which controls the effective time. For MNPs with diameter smaller than 15 nm, $\tau_{N}$ is smaller than $\tau_{B}$ and hence $\tau_{\mathrm{eff}}$ is dominated by $\tau_{N}$ [8]. On the other hand for NPs with diameter larger than 15 nm, $\tau_{B}$ is smaller than $\tau_{N}$ and hence $\tau_{\mathrm{eff}}$ is dominated by $\tau_{B}$ [8]. Both of these mechanisms contribute towards magnetic hyperthermia of magnetic NP. In obtaining Equations (3) and (4) it is assumed that the particles are identical (same size and shape) and non-interacting single-domain particles. In addition, Equations (1) and (2) are valid for zero applied magnetic field. If the applied field is not zero, then Zeeman energy should be included [9].

### 2.2. Power Dissipation in MNPs

The internal energy of a magnetic system in an adiabatic process is equal to the magnetic work done on it [22]:

(5) $$U=-\mu_{0}\oint^{}MdH$$

The power dissipation in the magnetic system, during a complete magnetic field cycle, is equal to internal energy divided by the time. Thus, the power dissipation, during several cycles, is equal to internal energy multiplied by the frequency:

(6) P=Uf

The power dissipated in a MNP due to the application of an alternating magnetic field of maximum strength H, frequency f ( ω=2πf ) was proposed to depend on magnetic spin relaxations of superparamagnetic NPs and is given by [22]:

(7) $$P\left(f,\;H\right)=Uf=\;{\pi \mu }_{0}\chi^{\prime \prime }H^{2}f$$

where $\mu_{0}$ is the permeability of free space and $\chi^{\prime \prime }$ is the imaginary part of the susceptibility χ ($\chi =\chi^{'}-i\chi^{\prime \prime }$). In the linear response theory (LRT), χ is assumed to remain constant with increasing H (M=χH). This approach was shown to be valid for very small magnetic fields. To be more specific, the LRT is valid in the superparamagnetic regime where $H_{\max}<k_{B}T/\mu_{0}M_{s}V$ and when the magnetization of NPs is linearly proportional to the applied magnetic field. This means that the applied fields should be much smaller than the saturation field of the NPs ($H_{\max}\ll H_{K}$) where $H_{\max}$ is the amplitude of the alternating applied magnetic field and $H_{K}$ is the anisotropy field [16]. The imaginary part of the susceptibility, $\chi^{\prime \prime }$ is given by [23,24]:

(8) $$\chi^{\prime \prime }=\frac{\omega \tau }{1+{\left(\omega \tau \right)}^{2}}\chi_{0}$$

(9) $$\chi_{0}=\frac{\mu_{0}M_{s}^{2}V}{k_{B}T}$$

where τ is the effective magnetic relaxation time, V is the volume of the NP, and $M_{s}$ is the saturation magnetization.

The heating efficiency is represented by the specific loss power (SLP) also referred to as the specific absorption rate (SAR). The specific loss power (SLP), which is measured in watts per gram, is given by [25]:

(10) $$SLP\left(f,H\right)=\frac{P\left(f,\;H\right)}{\rho }=\frac{{\pi \mu }_{0}\chi^{\prime \prime }H^{2}f}{\rho }$$

where ρ is the mass density of the magnetic material.

In living organs, the water-based medium around the cells absorb a lot of the heat generated by the NPs. Hence, for MNPs to have practical medical applications they should generate large SLP. It is clear that in the Rosensweig’s theory or LRT, the heat dissipation of the MNPs depends on several factors such as: strength and frequency of the applied magnetic field, the solvent viscosity, the size of the particles, the saturation magnetization and the magnetic anisotropy of the MNP. The strength and frequency of the applied magnetic field cannot have any value for applications on living organs. It is well-known that eddy currents are induced in a conductor due to an alternating magnetic field. These currents cause heating in the conductor. In human body, water is a conductor and hence eddy currents can be induced in the body under an alternating magnetic field which could damaging effect. Hence, there must be a criterion which imposes an upper limit for the allowed magnetic field that can be applied to living organs. The allowed frequency and amplitude of the alternating magnetic field that can be considered safe is not completely agreed on. In [26], the authors discussed the origins of these safety limits and pointed out they are self-imposed limits which are the subject of some debate. These safety limits were based on the work of Atkinson in 1984 [27] who performed some clinical tolerance tests on a healthy volunteers. He conducted the test using a single-turn induction coil which was placed around the thorax of the volunteer. Atkinson found that field intensities up to 35.8 A·turns/m at a frequency of 13.56 MHz can be thermally tolerated for extended periods of time [27]. This clinical tolerance is not known to be repeated [26] and the results reported by Atkinson become accepted as a safety limit which is known now as “Brezovich criterion” [28] where the product C=H·f should not exceed $4.5\times 10^{8}\;{\mathrm{Am}}^{-1}s^{-1}$. The Brezovich criterion is considered at best an upper limit for H·f when applying a uniform field over an entire thorax of an adult [26]. In practice, smaller coils are used with inhomogeneous fields and off-axis field directions which are significantly different conditions than those used by Atkinson. These factors are expected to reduce eddy current heating. In addition to that clinical tolerability to counteract cancer is expected to be higher than that of a healthy volunteer. Hence, the Brezovich criterion should not be considered as the only criterion. In [25], the authors suggested another criterion $C=H\cdot f=5\times 10^{9}\;{\mathrm{Am}}^{-1}s^{-1}$ which is 10 times larger than the Brezovich criterion. Hence, the two criteria for the product of the amplitude and the frequency (H·f) of the applied magnetic field are 4.85 × 10^8^ Am^−1^ s^−1^ (6 × 10^6^ Oe Hz) [26] and 5 × 10^9^ Am^−1^ s^−1^ (6.25 × 10^7^ Oe Hz) [25]. When the frequency is fixed at 100 kHz (which is very suitable for medical applications) *H* will be between 4.85 × 10^3^ Am^−1^ (60 Oe) and 50 × 10^3^ Am^−1^ (625 Oe) [29].

As mentioned above, the LRT is valid for very small applied fields. Hence, LRT will not be applicable for MNPs with low anisotropy energies where the magnetization is saturated at low applied fields. The alternative is to use Stoner-Wohlfarth model. However, the standard Stoner-Wohlfarth model is applied when T=0 or in the limit of infinite frequency. Because thermal activation is not involved, the magnetization can switch direction between the two equilibrium positions (potential wells) by removing the energy barrier using an applied magnetic field. Thus, a modification of Stoner-Wohlfarth model which takes into account the thermal activation of magnetization and the sweeping rate of the alternating magnetic field was investigated in [16]. There, the role of finite temperature and frequency on the coercive field and areas of hysteresis loops, were studied using Stoner-Wohlfarth based theories. Analytical formulas for temperature and frequency dependent coercive field as well as for temperature and frequency dependent hysteresis loop area. A time-dependent magnetic field $H(t)=H_{\max}\cos(\omega t)$ that is applied to the MNP along a direction that makes an angle ϕ with respect to the easy axis. The authors used a two-level approximation where the thermally activated reversals of moments occur between the metastable points ($\theta_{1}$, $E_{1}$) and ($\theta_{2}$, $E_{2}$) across the saddle point ($\theta_{3}$, $E_{3}$). They then calculated the time dependence of the probabilities of the magnetization being in the first and second potential well, *p*_1_ and $p_{2}=(1-p_{1})$ respectively. The magnetization was calculated using the equation:

(11) $$M=M_{s}\left(p_{1}{\mathrm{cos\theta}}_{1}+(1-p_{2}){\mathrm{cos\theta}}_{2}\right)$$

where θ is the angle between the magnetization and the easy axis. The authors conducted a large number of simulations to investigate the dependence of the coercivity field on the frequency of the magnetic field. For the case with ϕ=0 (magnetic field is aligned along the easy direction), the results in [16] have good agreement with the results in [30] for κ smaller than 0.5 where they obtained the following equation:

(12) $$\mu_{0}H_{C}=\mu_{0}H_{K}\left(1-\kappa^{1/2}\right)$$

For the random orientation case, the authors obtained the following equation:

(13) $$\mu_{0}H_{C}=0.48\mu_{0}H_{K}\left(b-\kappa^{n}\right)$$

where *b* = 1 and *n* = 0.8 ± 0.05, $H_{K}$ is the anisotropy field, and κ is a dimensionless parameter for the variation of $H_{C}$ that includes temperature and takes into account the sweeping rate of the magnetic field. The authors then conducted simulations to investigate the frequency and temperature dependence of the hysteresis loop area. The authors also deduced suitable formulas to calculate the areas of major hysteresis loops using Stoner-Wohlfarth based theories. It is worth mentioning that in [16], the easy axis of MNPs were considered to be fixed since rotation of the whole MNP was not considered in the analytical estimates [31,32].

In [32,33], the authors used numerical simulations to discuss the dynamics of rotatable superparamagnetic and ferromagnetic NPs in aqueous phase resembling the cytoplasm in a large alternating magnetic field. The authors considered monodisperse spheroidal magnetite nanoparticles with non-magnetic surfactant layers. Hence, the dipolar interactions among NPs were neglected and the NPs are considered to be uniformly dispersed and do not aggregate. In the calculations, the crystalline, and surface anisotropy energies were neglected compared to the uniaxial shape anisotropy. The authors used a two-level approximation, which considers thermally activated reversals between two meta-stable directions via a midway saddle point. In the simulation of reversal and rotation, Brownian dynamics simulation were considered where the inertia of the nanoparticle were neglected. The results of this numerical study could not be explained by the conventional models that consider a linear response of thermodynamic equilibrium states ($H_{0}=0\;,\;T\neq 0$) or magnetic field-driven reversals ($H_{0}\neq 0\;,\;T=0$). For rotatable superparamagnetic NPs, the relaxation loss was found to have two maxima; the primary one which is attributed to the rapid Neel relaxation, and a secondary one which is attributed to the slow rotation of the magnetic easy axis of each nanoparticle in the large field. For the rotatable ferromagnetic NPs, due to high-frequency alternating magnetic field, longitudinal and planar orientations were formed, irrespective of the free energy, as dissipative structures.

In magnetic hyperthermia, heating of MNPs mostly occurs in a liquid medium. In the LRT, the magnetic response of MNPs in a liquid is assumed to be characterized by the effective magnetic relaxation time, $\tau_{\mathrm{eff}}$ (Equation (4)). However, the conventional LRT does not take into account the complex dynamics of MNPs in in a viscous liquid in an alternating external magnetic field of finite amplitude and hence it oversimplifies the real situation [34]. In [34], magnetic dynamics of an assembly of NPs dispersed in a viscous Liquid were theoretically studied using stochastic equations of motion. In this method, stochastic equations of motion were constructed and solved for two unit vectors; the unit magnetization vector and the director which is a unit vector that determines the space orientation of a MNP with uniaxial anisotropy. Two regimes of the stationary magnetization oscillations were obtained, depending on the amplitude of the alternating magnetic field. In the viscous regime, which occurs for low magnetic field amplitudes, $H_{0}\ll H_{k}$, the two unit vectors move in unison and out of phase with respect to the phase of the alternating magnetic field. In the magnetic regime, which occurs for $H_{0}\geq H_{k}$, the director oscillates slightly, while the unit magnetization vector jumps between the directions along and opposite to the direction of the external magnetic field. The transition between the oscillation regimes occurs within the range $0.5H_{k}\leq H_{0}<H_{k}$, depending on the magnetic field frequency and the liquid viscosity. The authors described the behavior of the low-frequency hysteresis loops as a function of the liquid viscosity and the amplitude and frequency of alternating magnetic field. The authors showed that SAR of an assembly of MNPs in a liquid can be significantly increased by selecting a suitable mode of magnetization oscillations. The authors reported that for an assembly of MNPs in viscous liquid, large SAR can be obtained in the intermediate excitation regime, $H_{0}\approx 0.5H_{k}$. For magnetic parameters typical for iron oxides, and for *H*_0_ = 200–300 Oe, and *f* = 300–500 kHz, the estimated SAR values can be of the order of 1 kW/g. The results of this paper [34] clearly show that the magnetic dynamics (for low frequency hysteresis loops) of NPs dispersed in a viscous liquid is significantly different from the behavior of NPs immobilized in a solid matrix.

In [35], the authors studied superparamagnetic particles, with uniaxial anisotropy, suspended in a viscous fluid and subjected to an alternating magnetic field. Both dissipation mechanisms; the internal (Néel) and the external (Brownian) magnetic relaxations were considered. The authors obtained simple expression for the dynamic susceptibility that takes into account both dissipation mechanisms. The energy absorption was compared to the conventional approach using a model polydisperse colloid containing maghemite nanoparticles. The viscous losses due to particle motion in the fluid were found to have important contribution to the full magnetic response of the particles and thus to the specific loss power. The authors suggested a modification to the conventional LRT where the field-independent Brownian relaxation time $\tau_{B}$ should be replaced by a field-dependent Brownian relaxation time $\tau_{B}(\mu H/k_{B}T)$ [36].

### 2.3. The Role of Anisotropy on Heating Efficiency

According to the LRT it is clear that the anisotropy is an important factor in enhancing the Néel relaxation time (Equation (2)). However, it should be emphasized that from Equations (8) and (10) the SLP is maximized when ωτ=1. This means that the increase of relaxation time does not always yield an increase in SLP. The frequency of the applied magnetic field must be correlated with the relaxation time such that ωτ=1 [8]. Hence increasing the anisotropy results in an increase in the relaxation time and allows for the use of lower frequencies of the magnetic field. In [16], the authors concluded that the anisotropy of MNPs is a key parameter in tuning magnetic hyperthermia. They suggested that magnetic anisotropy should become central in the experimental investigations of magnetic hyperthermia. However, it is important to realize that depending on other factors, the anisotropy can increase or decrease heating efficiency of NPs. For example, the authors in [16] reported that heating efficiency was maximized in low anisotropy ferromagnetic Fe NPs.

## 3. Types of Anisotropies

Most magnetic materials contain some type of anisotropy that affects their magnetic behavior [18]. The most common types of anisotropy are: (a) magnetocrystalline anisotropy (or magnetic anisotropy or crystalline anisotropy); (b) surface anisotropy; (c) shape anisotropy; (d) exchange anisotropy; and (e) induced anisotropy (for example, by stress). All these anisotropies have influence on the magnetic properties to certain extent. In nanoparticles, shape anisotropy and magnetocrystalline anisotropy are the most important. Magnetocrystalline anisotropy arises from spin-orbit interaction and energetically favors alignment of the magnetic moments along a specific crystallographic direction called the easy axis of the material. The magnetocrystalline anisotropy depends on the type of material, temperature and impurities and is independent of the sample shape and size. Shape anisotropy causes magnetization to depend on the shape of the sample. The magnetization of a long thin needle shaped sample is easier along its long axis compared with that along any of its short axes. For nanoparticles, shape anisotropy is the dominant form of anisotropy. Stress anisotropy implies that magnetization might change with stress. It was shown that magnetic anisotropy changes when the surfaces are modified or adsorb different molecules [37]. This means that surface structure significantly influence the magnetic anisotropy. Hence, due to their large ratio of surface to bulk atoms, the surface anisotropy of nanoparticles could be more significant than both the crystalline and shape anisotropy. Coating of nanoparticles can have an influence on their magnetic anisotropies and hence on their magnetic properties [38,39]. In this work we focus on surface and exchange anisotropies.

### 3.1. The Role of Exchange Anisotropy in Core-Shell Nanoparticles

In 1956, Meiklejohn and Beans discovered that the hysteresis loop of a sample of ferromagnetic cobalt (Co) nanoparticles that is surrounded by antiferromagnetic oxidized layer (CoO) was shifted along the field (horizontal) axis after cooling in an applied magnetic field, *H* [40,41]. An increase in coercivity, *H*_C_ is usually observed with the shift of hysteresis loop [41,42]. This new effect is called exchange bias effect and the amount of the horizontal loop shift is called the exchange bias field, *H*_EB_. This new type of magnetic anisotropy is called the exchange anisotropy or exchange coupling. The exchange anisotropy is suggested to be due to the interaction between the antiferromagnetic and the ferromagnetic materials. The exchange coupling could occur at the core-shell interface of different magnetic phases such as at an interface of a FM and AFM or FIM [42]. Some researchers suggested that the existence of pinned uncompensated spins in the AFM shell [43,44] or at the interface between the FM core and the AFM shell [43,44,45] could be the source of the exchange bias coupling. Several experimental studies showed the existence of uncompensated spins [46,47,48,49,50] but with orientations relative to the ferromagnetic magnetization. A satisfactory understanding of the mechanism of the exchange anisotropy of core-shell NPs at the microscopic level is not achieved yet [51]. In [52,53], core (FIM)-shell (AFM) Mn_3_O_4_–MnO and Mn_3_O_4_–Mn NPs it was found that the atomic structure and strain at the interface determine the interfacial exchange coupling. In [54] microstructural properties of core (AFM) MnO-shell (FM) Mn_3_O_4_ NPs were investigated. This arrangement is opposite the usual FM-AFM core-shell arrangement. The interface was found to be ordered, implying a strong interfacial coupling. At temperatures below *T*_C_ of the FM Mn_3_O_4_ shell, large exchange field (*H*_EB_) values were obtained. In [45], the exchange coupling (or exchange anisotropy) at the core (AFM)-shell (FIM) interface of FeO–Fe_3_O_4_ NPs determines *H*_C_ and *H*_E_. Large effective interface area reults in large interface exchange anisotropy which leads to large values of *H*_C_ and *H*_E_. In [52,53], defects at the core-shell interface resulted in a small interfacial exchange coupling. The interfacial defects were suggested to produce interfacial uncompensated spins. In [55], FePt (FM)–Fe_3_O_4_ (FIM) core-shell NPs were investigated. The intimate contact between the FePt core and Fe_3_O_4_ shell was reported to lead to an effective interface exchange coupling. Hence, tailoring of the magnetic properties of these NPs can be achieved by controlling the core-shell dimensions, and by varying the material parameters of the core and shell. In an interesting study [56], CoFe_2_O_4_ (core)–MnFe_2_O_4_ (shell) MNPs were investigated. The diameter of the CoFe_2_O_4_ core was 9 mm and the thickness of the MnFe_2_O_4_ shell was 3 nm. The coercivity, *H*_C_ was found to have values between those for the core and shell materials which reflects the magnetic coupling of the core-shell structure. The SLP of the core-shell structures were found to have nearly one order of magnitude larger than those of NPs of the core or shell materials alone. In addition to an enhanced SLP, the SLP was found to vary by varying the core or shell structure. It is suggested that the exchange coupling at the interface can be tuned to produce effective anisotropy, *K* and magnetization that could lead to enhanced SLP.

These studies show that the dimensions and structure of core-shell interface have an impact on the interface exchange anisotropy and hence, on the effective anisotropy of the core-shell NPs. Thus, by tuning the core–shell parameters, the effective anisotropy can be varied to result in large heating efficiency of core–shell MNPs.

### 3.2. Surface Anisotropy in Nanoparticles

Because of the increased ratio of surface atoms to core atoms in NPs, surface effects were suggested to have significant role on the properties of NPs. Surface effects in MNPs include lattice relaxation [57], charge transfer [58,59], oxidation [60], surface spin disorder which results in spin-glass-like structures and spin canting [61]. These effects and others could the cause of several observed magnetic properties of MNPs such as the enhancement of magnetic anisotropy [37,62,63,64] and the reduction in saturation magnetization [61]. The total magnetization of a MNP has two contributions; the magnetization due to the surface spins and the magnetization due to the core. The magnetization of the surface is suggested to be due to the surface effects [65]. Surface spin disorder was reported to occur in iron oxide nanoparticles and was though to lead to extremely high magnetic anisotropy [66,67]. Some of these observations were explained initially in terms of a dead magnetic layer at the surface of the NP [68]. However, others attributed these observations to the disordered surface spins that freeze in a spin glass-like state or to surface spin canting [61,69,70]. A frozen disordered surface spin structures could make it difficult to attain saturation even under the application of high magnetic fields [71,72]. It was suggested also that exchange bias effect occurs between the surface and core spins of antiferromagnetic NPs and resulted in shifts in the magnetization hysteresis loops [73]. Using molecular dynamic modeling, non-uniform strains in the surface layers with an average expansion of a few percent compared to bulk were predicted [74]. A stress-induced anisotropy field was suggested to result from this expansion. An increase in the effective magnetic anisotropy due to surface effects was reported in several studies [6,8,75,76].

With decreasing the size of NPs, the ratio of the number of surface atoms to that of the bulk atoms become larger yielding larger contribution of the surface magnetization. Hence, surface magnetic anisotropy is expected to contribute towards the total magnetic anisotropy of the MNP. The total magnetic anisotropy of MNPs that includes the contribution of surface and core of MNPs is given by the phenomenological expression [37,72,77]:

(14) $$K=K_{V}+\frac{6K_{S}}{D}$$

where $K_{V}$ is the magnetocrystalline anisotropy of the core, $K_{S}$ is the surface anisotropy of the particle, and D is the diameter of the particle (which is assumed to be spherical). Equation (14) has been used for its simplicity, but it might not be accurate to combine the surface anisotropy with the core anisotropy in this simple additive way. It is clear from Equation (14) that the surface contribution to the effective anisotropy increases with decreasing the size of the particle. Modified version of Equation (14) was proposed by some researchers [63].

The role of surface anisotropy on the efficiency of heating in magnetic hyperthermia was studied [78]. Single-domain cubic iron oxide particles were found to have superior magnetic heating efficiency compared to spherical particles of similar sizes. Using Monte Carlo simulations at the atomic level [78] cubic particles were reported to have larger surface anisotropy compared with the spherical particles. These results show the beneficial role of surface anisotropy in the improved heating power. These results demonstrate the importance of both the crystal quality and surface bond on the magnetic properties of the ferrimagnetic nanoparticles. However, it should be kept in mind that increasing the anisotropy does not always increase heating efficiency. For example, as mentioned earlier, heating power was maximized in large ferromagnetic NPs with low anisotropy [16].

## 4. The Role of Inter-Particle Interactions on the Heating Efficiency

Inter-particle dipolar interaction energy is proportional to 1/*r*^6^, where *r* is the inter-particle distance between particles. Hence, dipolar interactions between MNPs increases with decreasing the inter-particle distance when particle concentration increases. Dipolar interactions are expected to influence the magnetic relaxations of MNPs and thus influence their heating efficiency in the existence of an alternating magnetic field. Although the role of dipolar interactions on heating efficiency in magnetic hyperthermia is important, it is not well understood.

The role of magnetic interactions between magnetic nanoparticles on the heating efficiency for hyperthermia has been studied experimentally and theoretically [79,80,81,82,83]. In [17], the authors conducted numerical simulations and experiments on a system of Fe NPs of sizes between 5.5 and 28 nm. By comparing SAR from numerical simulations with those from experiments, the authors suggested that magnetic interactions decrease heating efficiency. In [83], the influence of magnetic interactions on magnetic hyperthermia efficiency was investigated by conducting SAR and high-frequency hysteresis loop measurements on systems of MNPs with the same size (with diameter around 13.5 nm) but with a varying anisotropy. Both kind of measurements were performed at the same frequency *f* = 54 kHz. The samples investigated were colloidal solutions composed of Fe, Fe*_x_*C*_y_*, Fe (core)–Fe*_x_*C*_y_* (shell), and FeCo nanoparticles. The anisotropy was varied by changing their composition. High-frequency hysteresis loops were measured at maximum applied magnetic field μ_0_*H*_max_ = 42 mT while in SAR measurements, it was varied between 0 and 60 mT. The authors suggested that the formation of chains of MNPs could be a key element to understand experimental data. They reported that the large particle–particle magnetic interactions (compared with the magneto-crystalline anisotropy) leads to the formation of chains of MNPs during hyperthermia experiments. The authors observed a correlation between the magnetic nanoparticle magnetocrystalline anisotropy and the squareness of their hysteresis loop in colloidal solution where particles with larger anisotropy displayed smaller squareness. The authors claimed that “*chains of MNPs with a uniaxial anisotropy are the only way to reach the maximum possible SAR with a given magnetic material*” [83]. These results could explain contradictory results in the literature on the influence of magnetic dipolar interactions on heating efficiency of MNPs for magnetic hyperthermia [84,85,86,87,88,89,90].

In an interesting work [91], the SAR of two series of aqueous magnetite (Fe_3_O_4_) NPs and polyacrylic acid (PAA)-coated Fe_3_O_4_ NPs based dispersions were investigated at different magnetite concentrations. Heat efficiency was found to decrease with magnetite concentration for the PAA–Fe_3_O_4_ NPs. On the other hand, the heating efficiency for the bare Fe_3_O_4_ NPs, was found to increase with increasing particle concentration. This behavior was attributed to dipolar interactions. It was suggested that with increasing NP concentration, dipolar interactions cause Neel relaxation times to increase resulting in decreasing SAR for the PAA–Fe_3_O_4_ NPs. The PAA coating also was suggested to change the hydrodynamic size of the particles and thus modifying the Brownian relaxation time. For the bare NPs it was suggested that dipolar interactions are significant even at low concentrations, while aggregations occur at high concentrations. This work summarizes the conflicting role MNP concentration on SAR.

In an excellent review [8], the authors tried to resolve these controversial reports where they calculated ωτ in some conflicting reports and suggested that MNP concentration always suppresses the relaxation time in all situations. They clarified that this reduction in relaxation time has opposite effects on SAR depending on whether the value of ωτ *>*1 or ωτ < 1. When ωτ < 1, SAR will decrease as the relaxation time, τ decreases while for ωτ *>*1, SAR will increase as τ decreases. Although this work [8] is very interesting and provide a coherent explanation of the conflicting experimental work, we have to emphasize that in the calculations of ωτ, the authors used the formulas for Néel and Brownian relaxation times which are known to be valid for identical and non-interacting particles. However, dipolar interactions were suggested to change the effective anisotropy of the particles [92]. In addition, the applied magnetic field strength should be in the linear region of the Langevin curve [93].

The effect of inter-particle dipolar interactions on heating efficiency was studied in [92]. The influence of particle chain formation was investigated on the heating efficiency. The experimental part of the study was conducted on low-anisotropy (spherical) as well as high-anisotropy (parallelepiped) ferrite-based magnetic fluids. It was found that heating efficiency decreases with increasing dipolar interactions. Using a theoretical model (which is valid for linear response regime) for dipole interactions it was found that in general dipolar interactions decrease heating efficiency. The theoretical model is based on the fact that dipolar interactions in linear chain arrangements increase the effective magnetic anisotropy. The authors suggested that several factors play roles in this process, such as particle size, chain size and experimental conditions, need to be optimized to enhance heating. It is important to mention that the anisotropy can increase or decrease heating efficiency of NPs depending on other experimental factors. For example, in [16], heating efficiency was maximized in low anisotropy ferromagnetic Fe NPs.

In [81], numerical investigation of the role of dipolar interactions on the hyperthermia efficiency was conducted. The authors reported that dipolar interactions decrease heating efficacy of MNPs. When studying different sample shapes the authors reported that hysteresis might slightly increase by small dipolar interactions.

In [94], two separate sets of agglomerated and dispersed (non-agglomerated) Fe_2_O_3_ ferrite NPs were studied. The heating efficiency of intensely agglomerated 15 nm Fe_2_O_3_ ferrite nanoparticles, was investigated in alternating magnetic field with frequency, *f* = 100 kHz and maximum strength, *H*_0_ = 13 kA/m. Although the inter-particle interactions are strong in such agglomerate, moderate SAR value was found. To determine the effect of the NP diameter on SAR, the authors also used a model which includes the dipolar interactions among MNPs in the agglomerate. Because the amplitude of the alternating magnetic field, *H*_0_ was considered to be comparable to the anisotropy field, *H*_K_, the model was based on the hysteresis losses that is valid for the non-linear region. Because of the large size of the agglomerates (hydrodynamic mean diameters larger than 200 nm), the mechanical movement of the particles in the fluid can be neglected. Hence, in the simulated model, Brown relaxations were neglected as a heat generation mechanism. For the dispersed sets of MNPs the authors showed that heating is dominated by Neel and Brown relaxations. The authors reported a clear dependence of SAR on MNP size in both the agglomerated and dispersed samples.

The inter- and intra-particle interactions were recently investigated in core–shell nanoparticles [95]. In that report [95] the authors reported the results of low-temperature magnetic measurements that were conducted on very small (3.3 nm in size) core-shell structured NPs. The core (MnFe_2_O_4_) was found to be well-ordered ferromagnetic. The shell (γ-Fe_2_O_3_) was found to display uniaxial anisotropy with disordered spins. The magnetic measurements were conducted on two NP samples; one with non-textured frozen dispersions and the other with disordered powder. The authors discussed three types of particle interactions; the dipolar, the intra-particle exchange bias, and the inter-particle exchange bias. The dipolar interaction is the magnetic interaction of the magnetic moments of the cores of the NPs. The intra-particle exchange bias interaction exists between the core and surface of each particle and results in horizontal shifts of the field-cooled hysteresis loops. The inter-particle exchange interaction exists between the surface spins of particles that are in contact with each other. The authors [95] found that for dilute frozen dispersions of NPs that are at a distance from each other (not in contact), the dipolar interactions and inter-particle exchange bias interactions are neglected while the intra-particle exchange bias interaction is the only existing interaction. On the other hand, in concentrated frozen dispersions of NPs at a distance, dipolar interactions become significant. In powder NPs that are in contact, the inter-particle exchange interactions were found to be dominant. This is an interesting study [95] since it allows for distinguishing between intra- and inter-particle exchange bias interactions by comparing the results of magnetic measurements on samples with non-textured frozen dispersions of NPs and powder NPs. These results enhances the knowledge about the factors that could contribute towards the heating efficiency of NPs.

In the interesting paper [96], a global view of the role of particle–particle magnetic interactions was given. To calculate hysteresis loops, the authors used a kinetic Monte–Carlo algorithm that correctly account for both time and temperature. SAR of MNPs dispersed inside spherical lysosomes was studied as a function of several parameters including volume concentration of the 20 nearest neighbors around a given NP. For large magnetic fields, magnetic interactions of NPs increase the coercive field, saturation field and hysteresis area of major loops. However, for small amplitude magnetic field such as those used in magnetic hyperthermia, the heating power as function of concentration can increase, decrease or display a bell shape, depending on the relationship between the applied magnetic field and the coercive/saturation fields of the NPs [96]. The volume concentration was found to strongly influence the heating properties of a given NP. Heating power was shown to be not homogeneous inside lysosomes and drastically changes with the position inside them. Hence, the local environment of a given NP was found to have a significant impact on its heating power. In certain conditions, the amplitude of variation of heating power with position inside lysosomes could be more than one order of magnitude. The NP diameter, anisotropy and the amplitude of the applied magnetic field were also found to significantly affect magnetic interactions.

## 5. Some Remarks about the Physics of Heating of Nanoparticles for Localized Magnetic Hyperthermia

### 5.1. The Role of Size Distribution

In determining the relaxation times and heat generated in MNPs, the particles were assumed to have the same size. However, in reality this assumption cannot be satisfied because usually there will be some size distribution of the NPs regardless of the synthesis method used. In practice, size distributions are broad and may extend from single domain to multi-domain NPs. Producing NP systems with sufficiently narrow size distribution, where only one defined reversal mechanism appears, is not a simple task. There are not enough studies to clearly understand the effect of the size distribution width on heating efficiency of NPs. In [97], the effect of size distribution of NPs (in the diameter range from 10 to 100 nm) on magnetic hysteresis losses was investigated using a phenomenological model. The authors derived an empirical expression for the dependence of hysteresis loss on field amplitude and particle size. It was shown that a useful choice of field amplitude and frequency depends strongly on the mean particle size and variance of the NPs that will be used for hyperthermia. The authors suggested that iron oxide NPs with narrow size distribution and with a mean diameter that corresponds to the maximum coercivity in the single domain size range could lead to maximum heating efficiency. Hence, in addition to the required narrow size distribution, the mean particle size should be adjusted in relation to the magnetic field amplitude in order to obtain maximum SLP. If the accurate mean particle size and the corresponding coercivity are not known, then the relation between the magnetic field amplitude and mean particle size might not be satisfied resulting in a situation where many particles will not be able to reach maximum SLP. In this case, the authors indicated that using NPs with a broader size distribution may be advantageous. In [22], polydispersity in the size distribution of MNPs was found to reduce the heating efficiency. When the size distribution was changed from highly monodisperse (σ = 0) to polydisperse (σ = 2.5) the heating rate was significantly decreased, where σ is standard deviation of the lognormal size distribution.

### 5.2. The Heating Curve

In magnetic hyperthermia experiments, an alternating magnetic field is applied to the nanoparticle sample and the variation of temperature is measured. The heating efficiency is represented by SAR or SLP is usually obtained from the initial slope of the measured data. This method ignores the entire heating curve and hence does not display the entire temperature dependence of SAR [98].

The authors in [99] discussed several analytical method that is used to obtain the SAR from calorimetric measurements and pointed out that SAR values depend also on the analytical method used. The commonly used “initial slope” method was found to sensitive to the experimental conditions and could underestimate values by up to 25%. The full-curve fit method was found to be better but also with underestimation by up to 10% can be expected. The “corrected slope” method which was derived by the authors [99] was found to be the most accurate method. The combined errors that associate the analytical methods with the experimental errors could lead to large variations between the actual SAR and the reported values.

### 5.3. Temperature Dependence of Saturation Magnetization and Magnetic Anisotropy

Usually, in magnetic hyperthermia models that are based on Rosensweig’s theory [22] magnetic properties such as the saturation magnetization and the anisotropy are considered to be constant with temperature. However these properties and others change significantly with temperature [100].

The saturation magnetization as function of temperature in bulk ferromagnetic or ferrimagnetic materials at low temperatures, is governed by the Bloch’s law [101]:

(15) $$M_{S\;}\left(T\right)=M\left(0\right)\left[1-{\left(\frac{T}{T_{0}}\right)}^{\alpha }\right]$$

Here, *T*_0_ is the temperature at which *M*_s_ becomes zero and *M*(0) is the saturation magnetization at 0 K. The Bloch’s exponent α = 3/2 for bulk materials. Bloch’s *T*^3/2^ law was based on magnon excitation of long wave-length spin-waves at low temperatures. However, due to finite size effects in nanoparticles, magnons could have wavelengths larger than the size of the particle leading to deviations from the *T*^3/2^ law. Several studies discussed the temperature dependence of the saturation magnetization in nanoparticles and reported deviations from Bloch’s law at low temperatures [102,103,104,105,106,107,108,109,110,111].

In the modified Bloch’s law for nanoparticles at intermediate temperatures, the Bloch’s exponent α was found to have values larger and smaller than 3/2 [10,112] and decays exponentially at low temperatures [106]. In [102], the temperature dependence of saturation magnetization in nickel ferrite nanoparticles was investigated where the surface spin and finite size effects in nanoparticles were found to have an important role. The deviations from Block’s law could be due to inter-particle interactions [113] and the size distribution of the particles, significant and the disordered surface spins which influence the surface anisotropy and hence the effective anisotropy in the particles.

The magnetic anisotropy is known to vary with temperature in bulk magnetic materials and was expected to change with temperature also in nanoparticles [114,115,116,117]. A theoretical investigation of the influence of temperature on the magneto-crystalline anisotropy in Fe, Co and Ni nanoparticles showed a clear decrease with increasing temperature [118]. The dependence of effective magnetic anisotropy on temperature was recently investigated in MnFe_2_O_4_ nanoparticles with cubic anisotropy [119]. The magnetic anisotropy was found to decrease significantly with increasing temperature. Surface effects, which are more pronounce in particles with cubic anisotropy, were suggested to play a role in small particles.

Using Monte Carlo simulations surface effects were also found to have a dominant factor in determining the effective anisotropy at low temperatures resulting in an overall cubic effective anisotropy even in spherical nanoparticles with uniaxial anisotropy [120]. The contribution of cubic anisotropy was found to decrease with increasing temperature faster than that in particles with uniaxial anisotropy. The effective magnetic anisotropy constant of Fe_2_O_3_ nanoparticles was found to decrease significantly with temperature [121].

### 5.4. Experimental and Theoretical Limitations in the Determination of SAR

There are several methods where SAR or SLP can be measure. These methods involves magnetic or calorimetric measurements. However, there are always some inaccuracies in these measurements and results should be carefully discussed. In a recent and interesting review [122] the sources of uncertainties of several available methods in measuring SAR were analyzed. Comparison between magnetic methods and calorimetric methods were also discussed. It was shown that inaccuracies in magnetic measurements mainly result from the lack of experimental set-ups that are needed for the application of suitable strength and frequency of the alternating magnetic field in magnetic hyperthermia experiments. Inaccuracies in SAR when using the calorimetric methods result mainly from the lack of matching between measuring conditions, thermal models, and experimental setups.

In [99], the authors indicated that when SAR are determined by calorimetric measurements, it is preferred to conduct the measurements under adiabatic conditions where external heat transfer is minimized. However, because it is difficult to build adiabatic measurement systems and because the measurements in these systems are time-consuming, the SAR measurements are mainly conducted in non-adiabatic systems which lead to in accurate results. The authors pointed out that by using suitable experimental and analytical methods, accurate SAR measurements can be made using non-adiabatic conditions, as long as heat losses from the non-adiabatic setup are accounted for. The paper also discussed the different ways of heat loss which are due to conduction, convection, radiation at high temperatures, and evaporating or melting of the sample. Then possible sources of the inaccuracy in SAR measurement were discussed. One of these is the spatial inhomogeneity of temperature in the sample which makes the location of the thermal probe in the sample important. Other source of error is the delaying of heating where it takes some time for the heating curve to take off after the start of the heating process. A third source of error is the change of heat capacity with temperature. A fourth source of error is the inhomogeneity of the magnetic field. In addition to those, peripheral heating, which is due to the experimental setup itself and is expected to vary in different laboratories depending on the system used.

An interesting paper [16] discussed the three types of theories that can be used for describing hysteresis loops of MNPs. These are: equilibrium functions, theories based on Stoner–Wohlfarth model, and the linear response theory (LRT). Limitations and domains of validity were discussed. The authors proposed that the separation between “relaxation losses” and “hysteresis losses” is artificial and not correct. The authors showed that the LRT is only pertinent for MNPs with strong anisotropy and for particles with small anisotropy, theories based on Stoner–Wohlfarth model should be used. The authors also stressed that LRT including Brownian motion is only valid for small magnetic field [36].

### 5.5. Self-Regulated Hyperthermia

Heating cells to temperatures between 42 and 46 °C (315–319 K) results in killing only tumor cells [5]. Above this temperature, healthy cells might be affected resulting in necrosis. Hence, it is preferred that the temperature of MNPs does not exceed this level. In practice, it is not simple to determine the temperature of cells in accurate manner during hyperthermia. Thus, having MNPs with Curie temperature, *T*_C_ above 42 °C and below 46 °C is essential for self-regulated hyperthermia. Once *T*_C_ is reached, the MNPs lose their magnetization. Hence, heating stops without the need to remove the external magnetic field. In order to have self-regulated hyperthermia it is essential to investigate new materials and structures of MNPs. Several studies reported partial success in this regard [123]. Here we discuss some of the recent work on controlling *T*_C_ of MNPs. In [124], the role of shape, size and composition on *T*_C_ of ferromagnetic NPs was theoretically investigated. It was found that reducing the particle size could result in a decrease of *T*_C_. The authors also investigated different ferromagnetic material compositions in combination with nonmagnetic materials such as Zn and Cu and magnetic ions such as Gd and Cr. It was suggested [124] that introducing these materials in the ferromagnetic material results in lower *T*_C_ due to the reduction of the exchange interaction between the magnetic ions in the NPs. In [125] Cu–Ni alloy NPs were found to have *T*_C_ in the range of 43–46 °C. In [126], manganese perovskite nanoparticles La_1−*x*_Sr*_x_*MnO_3_ with *x* = 0.25 with size in the range of 30–49 nm were found to have *T*_C_ near 352 K with large SAR values. This indicates that this material could be a good candidate for self-regulated hyperthermia. In [127], Curie temperatures of Mn_0.5_Zn_0.5_Gd*_x_*Fe_(2−*x*)_O_4_ ferrite nanoparticles, with *x* = 0, 0.5, 1.0, and 1.5 were investigated. *T*_C_ was found to vary with changing the Gd concentration indicating the possibility of tuning *T*_C_ of ferrite NPs. Doping Mn ferrite with Zn (Mn_1−*x*_Zn*_x_*O) and doping Zn ferrite with Gd (ZnGd*_x_*Fe_2−*x*_O_4_) was investigated to tune the Curie temperature in the range (42–43 °C) which is very suitable for hyperthermia applications [128]. In [129], the authors investigated the magnetic properties of Mn*_x_*Zn_1−*x*_Fe_2_O_4_ nanoparticles with Mn concentrations *x* = 0.8, 0.61, 0.5, and 0.2. They found that *T*_C_ increases with decreasing Mn concentration. Hence, the authors suggested that Curie temperature close to 42 °C (315 K) might be achieved by adjusting the Mn and Zn concentrations. In [130], magnetic measurements of Zn*_x_*Gd_1−*x*_Fe nanoparticles with *x* = 0.02, 0.05, 0.1, and 0.2 were conducted. The authors found that *T*_C_ has a nonmonotonic behavior with increasing *x*. All samples were found to have *T*_C_ larger than 665 K. The lowest *T*_C_ of 665 K (392 C) was found for the sample with *x* = 0.02. The authors suggested that larger Zn concentrations (0.2–0.5) might result in *T*_C_ in the range suitable for self-controlled hyperthermia. In [131] Ni_1−*x*_Cr*_x_* NPs were prepared by standard arc melting technique. Their magnetic properties were investigated using Vibrating Sample Magnetometer (VSM) and Superconducting Quantum Interference Device (SQUID) magnetometer. As the Cr concentration was increased from *x* = 4.54 wt% to *x* = 5.90 wt%, *T*_C_ was found to decrease almost linearly from 401 to 289 K. Hence Ni_1−*x*_Cr*_x_* NPs were found to be suitable material for self-regulating magnetic hyperthermia. In [132], chromium–nickel alloy (Cr*_x_*Ni_1−*x*_) NPs were prepared using water-in-oil microemulsion and mechanical milling and investigated for self-controlled magnetic hyperthermia. The *T*_C_ of the sample synthesized by microemulsion method (*x* = 20) was found to be 320 °C. For the series of NPs prepared by mechanical milling, some of the NPs (*x* = 26, 27, 28, 29) were found to have low *T*_C_ and some of them (*x* = 10, 15, 20) were found to have high *T*_C_. The NPs with *x* = 29, were found to have *T*_C_ of 43 °C and for NPs with *x* = 28, *T*_C_ was found to be 44 °C. As the Cr content (*x*) decreases, *T*_C_ was found to increase. The results in [131] and in [132] clearly revealed that *T*_C_ of Cr*_x_*Ni_1−*x*_ NPs can tuned by varying the synthesis method and by varying the Cr/Ni molar ratio.

## 6. Conclusions

The factors that influence local magnetic hyperthermia using magnetic nanoparticles were discussed. We have shown that surface anisotropy and core-shell interface anisotropy have a noticeable impact on the relaxation time and hence, could be tuned to enhance the heating efficiency of MNPs. The role of dipolar interactions was emphasized as an important factor in heating efficiency where the concentration of MNPs was found to suppress the relaxation time. We have shown that the role of size distribution of the particles was not well-investigated with contradictory results. The magnetic anisotropy and magnetization of MNPs were shown to decrease with temperature but not considered in most heating efficiency studies. Self-regulated hyperthermia was shown to have limited success.

## References

[1] Gilchrist R.K., Shorey W.D., Hanselman R.C., Parrott J.C., Taylor C.B. (1957). Selective inductive heating of lymph nodes. Ann. Surg..

[2] Jordan A., Scholz R., Wust P., Schirra H., Schiestel T., Schmidt H., Felix R. (1999). Endocytosis of dextran and silan-coated magnetite nanoparticles and the effect of intracellular hyperthermia on human mammary carcinoma cells *in vitro*. J. Magn. Magn. Mater..

[3] Moroz P., Jones S.K., Gray B.N. (2002). Magnetically mediated hyperthermia: Current status and future directions. Int. J. Hyperth..

[4] Laurent S., Dutz S., Häfeli U.O., Mahmoudi M. (2011). Magnetic fluid hyperthermia: Focus on superparamagnetic iron oxide nanoparticles. Adv. Colloid Interface Sci..

[5] Kumar C.S.S.R., Mohammad F. (2011). Magnetic nanomaterials for hyperthermia-based therapy and controlled drug delivery. Adv. Drug Deliv. Rev..

[6] Hergt R., Dutz S., Müller R., Zeisberger M. (2006). Magnetic particle hyperthermia: Nanoparticle magnetism and materials development for cancer therapy. J. Phys. Condens. Matter.

[7] Vallejo-Fernandez G., Whear O., Roca A.G., Hussain S., Timmis J., Patel V., O’Grady K. (2013). Mechanisms of hyperthermia in magnetic nanoparticles. J. Phys. D.

[8] Deatsch A.E., Evans B.A. (2014). Heating efficiency in magnetic nanoparticle hyperthermia. J. Magn. Magn. Mater..

[9] Dennis C.L., Ivkov R. (2013). Physics of heat generation using magnetic nanoparticles for hyperthermia. Int. J. Hyperth..

[10] Sun S., Murray C.B., Weller D., Folks L., Moser A. (2000). Monodisperse FePt nanoparticles and ferromagnetic FePt nanocrystal superlattices. Science.

[11] Rusponi S., Cren T., Weiss N., Epple M., Buluschek P., Claude L., Brune H. (2003). The remarkable difference between surface and step atoms in the magnetic anisotropy of two-dimensional nanostructures. Nat. Mater..

[12] Tartaj P., Morales M.D.P., Veintemillas-Verdaguer S., Gonz lez-Carreo T., Serna C.J. (2003). The preparation of magnetic nanoparticles for applications in biomedicine. J. Phys. D.

[13] Gupta A.K., Gupta M. (2005). Synthesis and surface engineering of iron oxide nanoparticles for biomedical applications. Biomaterials.

[14] Koksharov Y.A., Gubin S.P. (2009). Magnetism of Nanoparticles: Effects of Size, Shape, and Interactions. Magnetic Nanoparticles.

[15] Issa B., Obaidat I.M., Albiss B.A., Haik Y. (2013). Magnetic nanoparticles: Surface effects and properties related to biomedicine applications. Int. J. Mol. Sci..

[16] Carrey J., Mehdaoui B., Respaud M. (2011). Simple models for dynamic hysteresis loop calculations of magnetic single-domain nanoparticles: Application to magnetic hyperthermia optimization. J. Appl. Phys..

[17] Mehdaoui B., Meffre A., Carrey J., Lachaize S., Lacroix L.-M., Gougeon M., Chaudret B., Respaud M. (2011). Optimal size of nanoparticles for magnetic hyperthermia: A combined theoretical and experimental study. Adv. Funct. Mater..

[18] Guimarães A.P. (2009). Principles of Nanomagnetism.

[19] Néel L. (1949). Théorie du trainage magnétique des ferromagné tiques en grains fins avec applications aux terres cuites. Ann. Geophys..

[20] Brown W.F. (1963). Thermal fluctuations of a single-domain particle. Phys. Rev..

[21] Leslie-Pelecky D.L., Rieke R.D. (1996). Magnetic properties of nanostructured materials. Chem. Mater..

[22] Rosensweig R.E. (2002). Heating magnetic fluid with alternating magnetic field. J. Magn. Magn. Mater..

[23] Delaunay L., Neveu S., Noyel G., Monin J. (1995). A new spectrometric method, using a magneto-optical effect, to study magnetic liquids. J. Magn. Magn. Mater..

[24] Glöckl G., Hergt R., Zeisberger M., Dutz S., Nagel S., Weitschies W. (2006). The effect of field parameters, nanoparticle properties and immobilization on the specific heating power in magnetic particle hyperthermia. J. Phys. Condens. Matter.

[25] Hergt R., Dutz S. (2007). Magnetic particle hyperthermia—Biophysical limitations of a visionary tumour therapy. J. Magn. Magn. Mater..

[26] Ortega D., Pankhurst Q.A., O’Brien P. (2013). Magnetic Hyperthermia. Nanoscience, Volume 1: Nanostructures through Chemistry.

[27] Atkinson W.J., Brezovich I.A., Chakraborty D.P. (1984). Usable frequencies in hyperthermia with thermal seeds. IEEE Trans. Biomed. Eng..

[28] Brezovich I.A. (1988). Low frequency hyperthermia: Capacitive and ferromagnetic thermoseed methods. Med. Phys. Monogr..

[29] Kita E., Oda T., Kayano T., Sato S., Minagawa M., Yanagihara H., Kishimoto M., Mitsumata C., Hashimoto S., Yamada K. (2010). Ferromagnetic nanoparticles for magnetic hyperthermia and thermoablation therapy. J. Phys. D.

[30] Usov N.A., Grebenshchikov Y.B. (2009). Hysteresis loops of an assembly of superparamagnetic nanoparticles with uniaxial anisotropy. J. Appl. Phys..

[31] Usov N.A., Liubimov B.Y. (2012). Dynamics of magnetic nanoparticle in a viscous liquid: Application to magnetic nanoparticle hyperthermia. J. Appl. Phys..

[32] Mamiya H. (2013). Recent advances in understanding magnetic nanoparticles in AC magnetic fields and optimal design for targeted hyperthermia. J. Nanomater..

[33] Mamiya H., Jeyadevan B. (2011). Hyperthermic effects of dissipative structures of magnetic nanoparticles in large alternating magnetic fields. Sci. Rep..

[34] Usov N.A. (2010). Low frequency hysteresis loops of superparamagnetic nanoparticles with uniaxial anisotropy. J. Appl. Phys..

[35] Raikher Y.L., Stepanov V.I. (2014). Physical aspects of magnetic hyperthermia: Low-frequency AC field absorption in a magnetic colloid. J. Magn. Magn. Mater..

[36] Raikher Y.L., Stepanov V.I. (2008). Absorption of AC field energy in a suspension of magnetic dipoles. J. Magn. Magn. Mater..

[37] Bødker F., Mørup S., Linderoth S. (1994). Surface effects in metallic iron nanoparticles. Phys. Rev. Lett..

[38] Homola A., Lorenz M., Mastrangelo C., Tilbury T. (1986). Novel magnetic dispersions using silica stabilized particles. IEEE Trans. Magn..

[39] Hormes J., Modrow H., Bönnemann H., Kumar C.S.S.R. (2005). The influence of various coatings on the electronic, magnetic, and geometric properties of cobalt nanoparticles. J. Appl. Phys..

[40] Meiklejohn W.H., Bean C.P. (1956). New magnetic anisotropy. Phys. Rev..

[41] Meiklejohn W.H., Bean C.P. (1957). New magnetic anisotropy. Phys. Rev..

[42] Nogués J., Sort J., Langlais V., Skumryev V., Suriñach S., Muñoz J.S., Baró M.D. (2005). Exchange bias in nanostructures. Phys. Rep..

[43] Berkowitz A.E., Takano K. (1999). Exchange anisotropy—A review. J. Magn. Magn. Mater..

[44] Nogués J., Schuller I.K. (1999). Exchange bias. J. Magn. Magn. Mater..

[45] Sun X., Huls N.F., Sigdel A., Sun S. (2012). Tuning exchange bias in core/shell FeO/Fe_3_O_4_ nanoparticles. Nano Lett..

[46] Takano K., Kodama R.H., Berkowitz A.E., Diego S., Jolla L. (1997). Interfacial uncompensated antiferromagnetic spins: Role in unidirectional anisotropy in polycrystalline Ni_81_Fe_19_/CoO bilayers. Phys. Rev. Lett..

[47] Antel W.J., Perjeru F., Harp G.R. (1999). Spin structure at the interface of exchange biased FeMn/Co bilayers. Phys. Rev. Lett..

[48] Ohldag H., Regan T., Stöhr J., Scholl A., Nolting F., Lüning J., Stamm C., Anders S., White R. (2001). Spectroscopic identification and direct imaging of interfacial magnetic spins. Phys. Rev. Lett..

[49] Ohldag H., Scholl A., Nolting F., Arenholz E., Maat S., Young A., Carey M., Stöhr J. (2003). Correlation between exchange bias and pinned interfacial spins. Phys. Rev. Lett..

[50] Eimüller T., Kato T., Mizuno T., Tsunashima S., Quitmann C., Ramsvik T., Iwata S., Schütz G. (2004). Uncompensated spins in a micro-patterned CoFeB/MnIr exchange bias system. Appl. Phys. Lett..

[51] Kiwi M. (2001). Exchange bias theory. J. Magn. Magn. Mater..

[52] Si P.Z., Li D., Lee J.W., Choi C.J., Zhang Z.D., Geng D.Y., Brück E. (2005). Unconventional exchange bias in oxide-coated manganese nanoparticles. Appl. Phys. Lett..

[53] Si P.Z., Li D., Choi C.J., Li Y.B., Geng D.Y., Zhang Z.D. (2007). Large coercivity and small exchange bias in Mn_3_O_4_/MnO nanoparticles. Solid State Commun..

[54] Berkowitz A.E., Rodriguez G.F., Hong J.I., An K., Hyeon T., Agarwal N., Smith D.J., Fullerton E.E. (2008). Monodispersed MnO nanoparticles with epitaxial Mn_3_O_4_ shells. J. Phys. D.

[55] Zeng H., Sun S., Li J., Wang Z.L., Liu J.P. (2004). Tailoring magnetic properties of core/shell nanoparticles. Appl. Phys. Lett..

[56] Lee J.-H., Jang J.-T., Choi J.-S., Moon S.H., Noh S.-H., Kim J.-W., Kim J.-G., Kim I.-S., Park K.I., Cheon J. (2011). Exchange-coupled magnetic nanoparticles for efficient heat induction. Nat. Nanotechnol..

[57] Evans R., Nowak U., Dorfbauer F., Shrefl T., Mryasov O., Chantrell R.W., Grochola G. (2006). The influence of shape and structure on the Curie temperature of Fe and Co nanoparticles. J. Appl. Phys..

[58] Coey J.M.D., Wongsaprom K., Alaria J., Venkatesan M. (2008). Charge-transfer ferromagnetism in oxide nanoparticles. J. Phys. D.

[59] Cótica L.F., Santos I.A., Girotto E.M., Ferri E.V., Coelho A.A. (2010). Surface spin disorder effects in magnetite and poly(thiophene)-coated magnetite nanoparticles. J. Appl. Phys..

[60] Makhlouf S.A., Al-attar H., Kodama R.H. (2008). Particle size and temperature dependence of exchange bias in NiO nanoparticles. Solid State Commun..

[61] Kodama R.H. (1999). Magnetic nanoparticles. J. Magn. Magn. Mater..

[62] Luis F., Torres J.M., García L.M., Bartolomé J., Stankiewicz J., Petroff F., Fettar F., Maurice J.-L., Vaurès A. (2002). Enhancement of the magnetic anisotropy of nanometer-sized Co clusters: Influence of the surface and of interparticle interactions. Phys. Rev. B.

[63] Luis F., Bartolomé F., Petroff F., Bartolomé J., García L.M., Deranlot C., Jaffrès H., Martínez M.J., Bencok P., Wilhelm F. (2006). Tuning the magnetic anisotropy of Co nanoparticles by metal capping. Europhys. Lett..

[64] Lima E., de Biasi E., Mansilla M.V., Saleta M.E., Effenberg F., Rossi L.M., Cohen R., Rechenberg H.R., Zysler R.D. (2010). Surface effects in the magnetic properties of crystalline 3 nm ferrite nanoparticles chemically synthesized. J. Appl. Phys..

[65] Ho C.-H., Lai C.-H. (2006). Size-dependent magnetic properties of PtMn nanoparticles. IEEE Trans. Magn..

[66] Del Muro M.G., Batlle X., Labarta A. (1999). Erasing the glassy state in magnetic fine particles. Phys. Rev. B.

[67] Batlle X., del Muro M.G., Labarta A. (1997). Interaction effects and energy barrier distribution on the magnetic relaxation of nanocrystalline hexagonal ferrites. Phys. Rev. B.

[68] Berkowitz A.E., Schuele W.J., Flanders P.J. (1968). Influence of crystallite size on the magnetic properties of acicular-Fe_2_O_3_ particles. J. Appl. Phys..

[69] Batlle X., Pérez N., Guardia P., Iglesias O., Labarta A., Bartolomé F., Garcıa L.M., Bartolomé J., Roca A.G., Morales M.P. (2011). Magnetic nanoparticles with bulk-like properties. J. Appl. Phys..

[70] Bedanta S., Kleemann W. (2009). Supermagnetism. J. Phys. D.

[71] Papaefthymiou G.C. (2009). Nanoparticle magnetism. Nano Today.

[72] Ahmed S.R., Ogale S.B., Papaefthymiou G.C., Ramesh R., Kofinas P. (2002). Magnetic properties of CoFe_2_O_4_ nanoparticles synthesized through a block copolymer nanoreactor route. Appl. Phys. Lett..

[73] Park T.-J., Papaefthymiou G.C., Viescas A.J., Moodenbaugh A.R., Wong S.S. (2007). Size-dependent magnetic properties of single-crystalline multiferroic BiFeO_3_ nanoparticles. Nano Lett..

[74] Nunes A.C., Yang L. (1998). Calculated ferrite nanocrystal relaxation and its magnetic implications. Surf. Sci..

[75] Jamet M., Wernsdorfer W., Thirion C., Mailly D., Dupuis V., Mélinon P., Pérez A. (2001). Magnetic anisotropy of a single cobalt nanocluster. Phys. Rev. Lett..

[76] Jamet M., Wernsdorfer W., Thirion C., Dupuis V., Mélinon P., Pérez L., Mailly D. (2004). Magnetic anisotropy in single clusters. Phys. Rev. B.

[77] Pérez N., Guardia P., Roca A.G., Morales M.P., Serna C.J., Iglesias O., Bartolomé F., García L.M., Batlle X., Labarta A. (2008). Surface anisotropy broadening of the energy barrier distribution in magnetic nanoparticles. Nanotechnology.

[78] Martinez-Boubeta C., Simeonidis K., Makridis A., Angelakeris M., Iglesias O., Guardia P., Cabot A., Yedra L., Estradé S., Peiró F. (2013). Learning from nature to improve the heat generation of iron-oxide nanoparticles for magnetic hyperthermia applications. Sci. Rep..

[79] Burrows F., Parker C., Evans R.F.L., Hancock Y., Hovorka O., Chantrell R.W. (2010). Energy losses in interacting fine-particle magnetic composites. J. Phys. D.

[80] Gudoshnikov S.A., Liubimov B.Y., Usov N.A. (2012). Hysteresis losses in a dense superparamagnetic nanoparticle assembly. AIP Adv..

[81] Haase C., Nowak U. (2012). Role of dipole-dipole interactions for hyperthermia heating of magnetic nanoparticle ensembles. Phys. Rev. B.

[82] Martinez-Boubeta C., Simeonidis K., Serantes D., Conde-Leborán I., Kazakis I., Stefanou G., Peña L., Galceran R., Balcells L., Monty C. (2012). Adjustable hyperthermia response of self-assembled ferromagnetic Fe-MgO core-shell nanoparticles by tuning dipole-dipole interactions. Adv. Funct. Mater..

[83] Mehdaoui B., Tan R.P., Meffre A., Carrey J., Lachaize S., Chaudret B., Respaud M. (2013). Increase of magnetic hyperthermia efficiency due to dipolar interactions in low-anisotropy magnetic nanoparticles: Theoretical and experimental results. Phys. Rev. B.

[84] Hansen M.F., Mørup S. (1998). Models for the dynamics of interacting magnetic nanoparticles. J. Magn. Magn. Mater..

[85] Urtizberea A., Natividad E., Arizaga A., Castro M., Mediano A. (2010). Specific absorption rates and magnetic properties of ferrofluids with interaction effects at low concentrations. J. Phys. Chem. C.

[86] Dennis C.L., Jackson A.J., Borchers J.A., Ivkov R., Foreman A.R., Lau J.W., Goernitz E., Gruettner C. (2008). The influence of collective behavior on the magnetic and heating properties of iron oxide nanoparticles. J. Appl. Phys..

[87] Mørup S., Tronc E. (1994). Superparamagnetic relaxation of weakly interacting particles. Phys. Rev. Lett..

[88] Dormann J., Fiorani D., Tronc E. (1999). On the models for interparticle interactions in nanoparticle assemblies: Comparison with experimental results. J. Magn. Magn. Mater..

[89] Dormann J., D’Orazio F., Lucari F., Tronc E., Prené P., Jolivet J., Fiorani D., Cherkaoui R., Noguès M. (1996). Thermal variation of the relaxation time of the magnetic moment of γ-Fe_2_O_3_ nanoparticles with interparticle interactions of various strengths. Phys. Rev. B.

[90] Mørup S., Hansen M.H., Frandsen C. (2010). Magnetic interactions between nanoparticles. Beilstein J. Nanotechnol..

[91] Piñeiro-Redondo Y., Bañobre-López M., Pardiñas-Blanco I., Goya G., López-Quintela M.A., Rivas J. (2011). The influence of colloidal parameters on the specific power absorption of PAA-coated magnetite nanoparticles. Nanoscale Res. Lett..

[92] Branquinho L.C., Carrião M.S., Costa A.S., Zufelato N., Sousa M.H., Miotto R., Ivkov R., Bakuzis A.F. (2013). Effect of magnetic dipolar interactions on nanoparticle heating efficiency: Implications for cancer hyperthermia. Sci. Rep..

[93] Hergt R., Dutz S., Zeisberger M. (2010). Validity limits of the Néel relaxation model of magnetic nanoparticles for hyperthermia. Nanotechnology.

[94] Lima E., de Biasi E., Mansilla M.V., Saleta M.E., Granada M., Troiani H.E., Effenberger F.B., Rossi L.M., Rechenberg H.R., Zysler R.D. (2013). Heat generation in agglomerated ferrite nanoparticles in an alternating magnetic field. J. Phys. D.

[95] Silva F.G., Aquino R., Tourinho F.A., Stepanov V.I., Raikher Y.L., Perzynski R., Depeyrot J. (2013). The role of magnetic interactions in exchange bias properties of MnFe_2_O_4_@γ-Fe_2_O_3_ core/shell nanoparticles. J. Phys. D.

[96] Tan R.P., Carrey J., Respaud M. (2014). Magnetic hyperthermia properties of nanoparticles inside lysosomes using kinetic Monte–Carlo simulations: Influence of key parameters, of dipolar interactions and spatial variations of heating power. Phys. Rev. B.

[97] Hergt R., Dutz S., Röder M. (2008). Effects of size distribution on hysteresis losses of magnetic nanoparticles for hyperthermia. J. Phys. Condens. Matter.

[98] Landi G.T. (2013). Simple models for the heating curve in magnetic hyperthermia experiments. J. Magn. Magn. Mater..

[99] Wildeboer R.R., Southern P., Pankhurst Q.A. (2014). On the reliable measurement of specific absorption rates and intrinsic loss parameters in magnetic hyperthermia materials. J. Phys. D.

[100] Ondeck C.L., Habib A.H., Ohodnicki P., Miller K., Sawyer C.A., Chaudhary P., McHenry M.E. (2009). Theory of magnetic fluid heating with an alternating magnetic field with temperature dependent materials properties for self-regulated heating. J. Appl. Phys..

[101] Bloch F. (1930). Zur Theorie des Ferromagnetismus. Z. Phys..

[102] Maaz K., Mumtaz A., Hasanain S.K., Bertino M.F. (2010). Temperature dependent coercivity and magnetization of nickel ferrite nanoparticles. J. Magn. Magn. Mater..

[103] Della Torre E., Bennett L.H., Watson R.E. (2005). Extension of the Bloch T^3/2^ law to magnetic nanostructures: Bose-Einstein condensation. Phys. Rev. Lett..

[104] Senz V., Röhlsberger R., Bansmann J., Leupold O., Meiwes-Broer K.-H. (2003). Temperature dependence of the magnetization in Fe islands on W(110): Evidence for spin-wave quantization. New J. Phys..

[105] Hendriksen P.V., Linderoth S., Lindgard P.A. (1992). Finite-size effects in the magnetic properties of ferromagnetic clusters. J. Magn. Magn. Mater..

[106] Hendriksen P.V., Linderoth S., Lindgard P.A. (1993). Finite-size modifications of the magnetic properties of clusters. Phys. Rev. B.

[107] Linderoth S., Balcells L., Labarta A., Tejada J., Hendriksen P.V., Sethi S.A. (1993). Magnetization and Mössbauer studies of ultrafine Fe–C particles. J. Magn. Magn. Mater..

[108] Eggeman A.S., Petford-Long A.K., Dobson P.J., Wiggins J., Bromwich T., Dunin-Borkowski R., Kasam T. (2006). Synthesis and characterisation of silica encapsulated cobalt nanoparticles and nanoparticle chains. J. Magn. Magn. Mater..

[109] Alves C.R., Aquino R., Sousa M.H., Rechenberg H.R., Goya G.F., Tourinho F.A., Depeyrot J. (2004). Low temperature experimental investigation of finite-size and surface effects in CuFe_2_O_4_ nanoparticles of ferrofluids. J. Metastable Nanocryst. Mater..

[110] Mandal K., Mitra S., Kumar P.A. (2006). Deviation from Bloch T^3/2^ law in ferrite nanoparticles. Europhys. Lett..

[111] Morup S. (2007). Comment on “Deviation from the Bloch T^3/2^ law in ferrite nanoparticles” by K. Mandal *et al*.. Europhys. Lett..

[112] Ortega D., Vélez-Fort E., García D.A., García R., Litrán R., Barrera-Solano C., Ramírez-del-Solar M., Domínguez M. (2010). Size and surface effects in the magnetic properties of maghemite and magnetite coated nanoparticles. Phil. Trans. R. Soc. A.

[113] Gubin S.P., Koksharov Y.A., Khomutov G.B., Yurkov G.Y. (2005). Magnetic nanoparticles: Preparation, structure and properties. Russ. Chem. Rev..

[114] Fonseca F., Goya G., Jardim R., Muccillo R., Carreño N., Longo E., Leite E. (2002). Superparamagnetism and magnetic properties of Ni nanoparticles embedded in SiO_2_. Phys. Rev. B.

[115] Tronc E., Fiorani D., Noguès M., Testa A., Lucari F., D’Orazio F., Grenèche J., Wernsdorfer W., Galvez N., Chanéac C. (2003). Surface effects in noninteracting and interacting γ-Fe_2_O_3_ nanoparticles. J. Magn. Magn. Mater..

[116] Tronc E., Noguès M., Chanéac C., Lucari F., D’Orazio F., Grenèche J., Jolivet J., Fiorani D., Testa A. (2004). Magnetic properties of γ-Fe_2_O_3_ dispersed particles: Size and matrix effects. J. Magn. Magn. Mater..

[117] De Julián Fernández C., Mattei G., Sangregorio C., Battaglin C., Gatteschi D., Mazzoldi P. (2004). Superparamagnetism and coercivity in HCP-Co nanoparticles dispersed in silica matrix. J. Magn. Magn. Mater..

[118] De Julián Fernández C. (2005). Influence of the temperature dependence of anisotropy on the magnetic behavior of nanoparticles. Phys. Rev. B.

[119] Yoon S., Krishnan K.M. (2011). Temperature dependence of magnetic anisotropy constant in manganese ferrite nanoparticles at low temperature. J. Appl. Phys..

[120] Yanes R., Chubykalo-Fesenko O., Evans R.F.L., Chantrell R.W. (2010). Temperature dependence of the effective anisotropies in magnetic nanoparticles with Néel surface anisotropy. J. Phys. D.

[121] Yoon S. (2011). Determination of the temperature dependence of the magnetic anisotropy constant in magnetite nanoparticles. J. Korean Phys. Soc..

[122] Andreu I., Natividad E. (2013). Accuracy of available methods for quantifying the heat power generation of nanoparticles for magnetic hyperthermia. Int. J. Hyperth..

[123] Martirosyan K.S. (2012). Thermosensitive magnetic nanoparticles for self-controlled hyperthermia cancer treatment. J. Nanomed. Nanotechol..

[124] Apostolova I., Wesselinowa J.M. (2009). Possible low-*T*_C_ nanoparticles for use in magnetic hyperthermia treatments. Solid State Commun..

[125] Chatterjee J., Bettge M., Haik Y., Chen C.J. (2005). Synthesis and characterization of polymer encapsulated Cu–Ni magnetic nanoparticles for hyperthermia applications. J. Magn. Magn. Mater..

[126] Pollert E., Knížek K., Maryško M., Kašpar P., Vasseur S., Duguet E. (2007). New-tuned magnetic nanoparticles for self-controlled hyperthermia. J. Magn. Magn. Mater..

[127] Obaidat I.M., Mohite V., Issa B., Tit N., Haik Y. (2009). Predicting a major role of surface spins in the magnetic properties of ferrite nanoparticles. Cryst. Res. Technol..

[128] Apostolov A.T., Apostolova I.N., Wesselinowa J.M. (2011). MO.Fe_2_O_3_ nanoparticles for self-controlled magnetic hyperthermia. J. Appl. Phys..

[129] Hejase H., Hayek S.S., Qadri S., Haik Y. (2012). MnZnFe nanoparticles for self-controlled magnetic hyperthermia. J. Magn. Magn. Mater..

[130] Hejase H.A., Hayek S.S., Qadri S.M., Haik Y.S. (2012). Self-controlled hyperthermia characteristics of ZnGdFe nanoparticles. IEEE Trans. Magn..

[131] Akin Y., Obaidat I.M., Issa B., Haik Y. (2009). Ni_1−*x*_Cr*_x_* alloy for self-controlled magnetic hyperthermia. Cryst. Res. Technol..

[132] Ban I., Stergar J., Drofenik M., Ferk G., Makovec D. (2013). Synthesis of chromium-nickel nanoparticles prepared by a microemulsion method and mechanical milling. Acta Chim. Slov..

